# UTILIZATION TRENDS OF NEWLY APPROVED GLUCOSE-LOWERING DRUGS FOR TYPE 2 DIABETES

**DOI:** 10.1093/geroni/igz038.2155

**Published:** 2019-11-08

**Authors:** Chintan Dave, Dae Kim, Elisabetta Patorno

**Affiliations:** 1 Brigham and Women’s Hospital, Boston, Massachusetts, United States; 2 Hebrew SeniorLife, Boston, Massachusetts, United States

## Abstract

Using Medicare fee-for-service data from 2013-2015, we identified 3.2 million patients per year (mean [SD] age, 74.7 years [standard deviation, 7.2]) who were treated with glucose-lowering drugs for type 2 diabetes. Between 2013 and 2015, the proportion of patients treated with sulfonylureas declined from 27.4% to 25.1%; those using DPP4is (11.5% to 12.0%) and GLP1-RAs (1.8% to 2.4%) remained unchanged; those using SGLT2is increased from 0.2% to 1.9%. In the subgroup of patients initiating a glucose-lowering drug without prior use of the same class agent, the proportion of patients starting sulfonylureas (18.7% to 17.2% of initiators), DPP4is (16.0% to 15.0% of initiators), and GLP1-RAs (3.4% to 4.2% of initiators) changed little between 2013 and 2015, while those starting SGLT2is increased from 0.7% to 6.5% of initiators. In the Medicare population, we observed a persistently high use of sulfonylureas and a rapid uptake of SGLT2is among the newer classes.

